# Development and Pilot Evaluation of Mock American Board of Emergency Medicine Ultrasound Cases for Emergency Medicine Residents

**DOI:** 10.7759/cureus.115449

**Published:** 2026-08-30

**Authors:** Neil Wallace, David Wasiak, Martin Demant, Timothy Buckley, Ofek Belkin, Alexandra Oosting, Josie Acuña

**Affiliations:** 1 Emergency Medicine, Univeristy of Arizona College of Medicine, Tucson, USA; 2 Emergency Medicine, University of Arizona College of Medicine, Tucson, USA; 3 Emergency Medicine, Florida Atlantic University Charles E. Schmidt College of Medicine, Boca Raton, USA

**Keywords:** certifying examination, emergency medicine, medical education, point-of-care ultrasound, residency, ultrasound

## Abstract

Background: The maturation of point-of-care ultrasound (POCUS) within the specialty of Emergency Medicine (EM) has led to its incorporation into the newest iteration of the American Board of Emergency Medicine (ABEM) Certifying Examination. The new iteration of the exam will consist of ultrasound cases that will include at least two image acquisition and two interpretation components. Examinees will be asked to acquire and interpret ultrasound images on a standardized patient. The objective of this study was to evaluate resident performance and confidence during a two-case mock ultrasound curriculum designed to familiarize trainees with the ultrasound component of the ABEM Certifying Examination.

Methods: This is a single blinded, cross-over study at the University of Arizona College of Medicine (Tucson, Arizona, United States). The study participants were EM resident physicians. A panel of POCUS experts developed two mock cases modeled after the ABEM certification ultrasound case. Residents were randomly assigned to a cardiac or thoracic-based case. Prior to participation, they were asked to independently watch the ABEM instructional video. They underwent an examination with a standardized patient, proctored by POCUS experts. After a one-month interval, participants were assessed on the alternate case. Data were collected from January 1, 2025 to May 1, 2026.

Results: 64 EM residents participated in the study. 48 completed the cross-over design, and 16 completed one day of the examination. Those that did review the material scored higher with a median score of 14 compared to 13, however, this was not statistically significant (p-value of 0.36). On Day 1, the mean overall score (out of 15) was 11.96 ± 2.49 for the Cardiac group and 11.62 ± 1.92 for the Pulmonary group. On Day 2, the mean overall scores increased to 12.55 ± 2.45 and 12.75 ± 2.24, respectively. There was no significant difference in mean scores between the Cardiac and Pulmonary cases on either Day 1 (p=0.57) or Day 2 (p=0.74). When comparing performance across days within each case type, Pulmonary case scores demonstrated a statistically significant increase from Day 1 to Day 2 (p=0.05). Across all participants on Day 1, post-graduate year (PGY) level was moderately correlated with total score (r=0.45, p=0.0007), with more senior residents tending to achieve higher examination scores.

Conclusion: A focused, two-case preparatory curriculum was feasible across multiple residency programs and associated with differences in performance across testing days, particularly in the pulmonary ultrasound scenarios. Early exposure to mock examination cases may help residents develop familiarity with the observed structured clinical examination (OSCE)-style interface, strengthen technical and communication skills, and improve readiness for board certification.

## Introduction

Recent changes to the American Board of Emergency Medicine (ABEM) certifying exam have had a significant impact on the testing of point-of-care ultrasound (POCUS) skills. Ultrasound is now listed as a content area in the communication and procedural cases description. The new iteration of the exam will consist of POCUS cases that will include at least two image acquisition and two interpretation components. According to ABEM, the goal of the changes was to test additional competencies, such as bedside ultrasound, that were not addressed on the qualifying exam or the current version of the oral exam [1]. The latest version of the certifying exam, expected to begin in 2026, is completed in person and is composed of clinical, communication and procedural cases. Examinees will be asked to acquire and interpret ultrasound images on a standardized patient. They will be graded on their ability to obtain adequate ultrasound images, image interpretation, and the provision of patient-centered care.

The suggested core content and expected competencies for emergency medicine (EM) residents have been well described and refined for more than a decade [2,3]. However, the introduction of the new certifying examination format has created a critical need for targeted preparatory materials that align with these changes and help residents apply their ultrasound skills within the observed structured clinical examination (OSCE) setting. As with any new examination interface, it is important for examinees to become familiar with both the format and content. The transition to this updated examination therefore highlights the need for curriculum specifically designed to prepare EM residents for board certification. The objective of this study was to evaluate resident performance and confidence during a two-case mock ultrasound curriculum designed to familiarize trainees with the ultrasound component of the ABEM Certifying Examination.

## Materials and methods

This is a crossover study, conducted at the University of Arizona College of Medicine (Tucson, Arizona, United States). This institution has three Accreditation Council for Graduate Medical Education-accredited categorical three-year EM residency programs and a five-year combined EM and pediatric residency program. The emergency departments (EDs) at this institution have ongoing emergency ultrasound training programs. The study participants were EM resident physicians from various stages of their training who voluntarily chose to participate. All residents from the three residency training programs were invited to participate, and consent was obtained from all participants. Residents who declined participation were not enrolled. This study was approved by the institutional review board at the University of Arizona (Protocol Number: 1806663505).

A panel of POCUS experts developed two mock cases modeled after the ABEM certification ultrasound case. The experts were EM physicians, fellowship-trained in emergency ultrasound. The cases that were developed included materials for the examinee, a script including case parameters and ultrasound machine setting characteristics for the examiner, as well as scoring criteria. The panel of POCUS experts created the scoring criteria and met until a consensus was obtained on what criteria should be included. The scoring criteria components included patient-centered care, image acquisition, and image interpretation, which were further broken down into anatomy and application criteria, and representative ultrasound pathology clips or still images. The final criteria consisted of 15 critical actions for each case (Appendix 1,2). Completion of each action resulted in one point being awarded. Partial credit was not given.

Participants were randomized to begin with either the cardiac or the pulmonary case and crossed over to the alternate case following a one-month interval. Randomization was performed using Excel 365 (Microsoft Corporation, United States). Participants were assigned evenly to each sequence of cases. Allocation was concealed prior to the first examination. Prior to participation, participants were asked to independently watch the ABEM instructional video on the ultrasound portion of the certifying exam, however this was not required [4]. Participants self-reported use of the instructional video. Regardless of whether the participant had already watched the instructional video, they were given a brief review on the examination process. Participants then underwent an examination with a standardized patient. The standardized patients were employed and trained by the institution, and briefed by the examiners on the study plan and procedures. For these sessions, they were instructed to have minimal verbal interaction with the participant. They were allowed to reposition as requested by the participant. The POCUS experts proctored the exam and subsequently evaluated the residents. Participants were blinded to their initial case assignment prior to starting the examination and case outcomes. After a one-month period, they were provided a focused study guide of high-yield EM- focused POCUS topics, and then underwent the crossover examination to the alternate case by the same group of POCUS experts.

Case 1: Cardiac ultrasound

Case 1 was aimed at assessing the examinee’s ability to perform a cardiac ultrasound and assess a patient presenting with shortness of breath. The proctor was provided with a script (Appendix 3), which guided the resident to demonstrate how you would assess dyspnea using ultrasound to evaluate left ventricular function with a parasternal long-axis view. Residents were asked to state their probe of choice. When obtaining their images on the standardized patient, the examinees were prompted to ask the proctor to adjust gain and probe settings as needed. Examinees were asked to state when they felt they had obtained an acceptable parasternal long-axis view to evaluate for left ventricular function on the standardized patient and display for the proctor to assess. Examinees were asked to identify several anatomic structures on this image (Appendix 1). Examinees were then to direct your attention to an alternate screen in the room that displayed a video clip (Figure 1).

**Figure 1:**
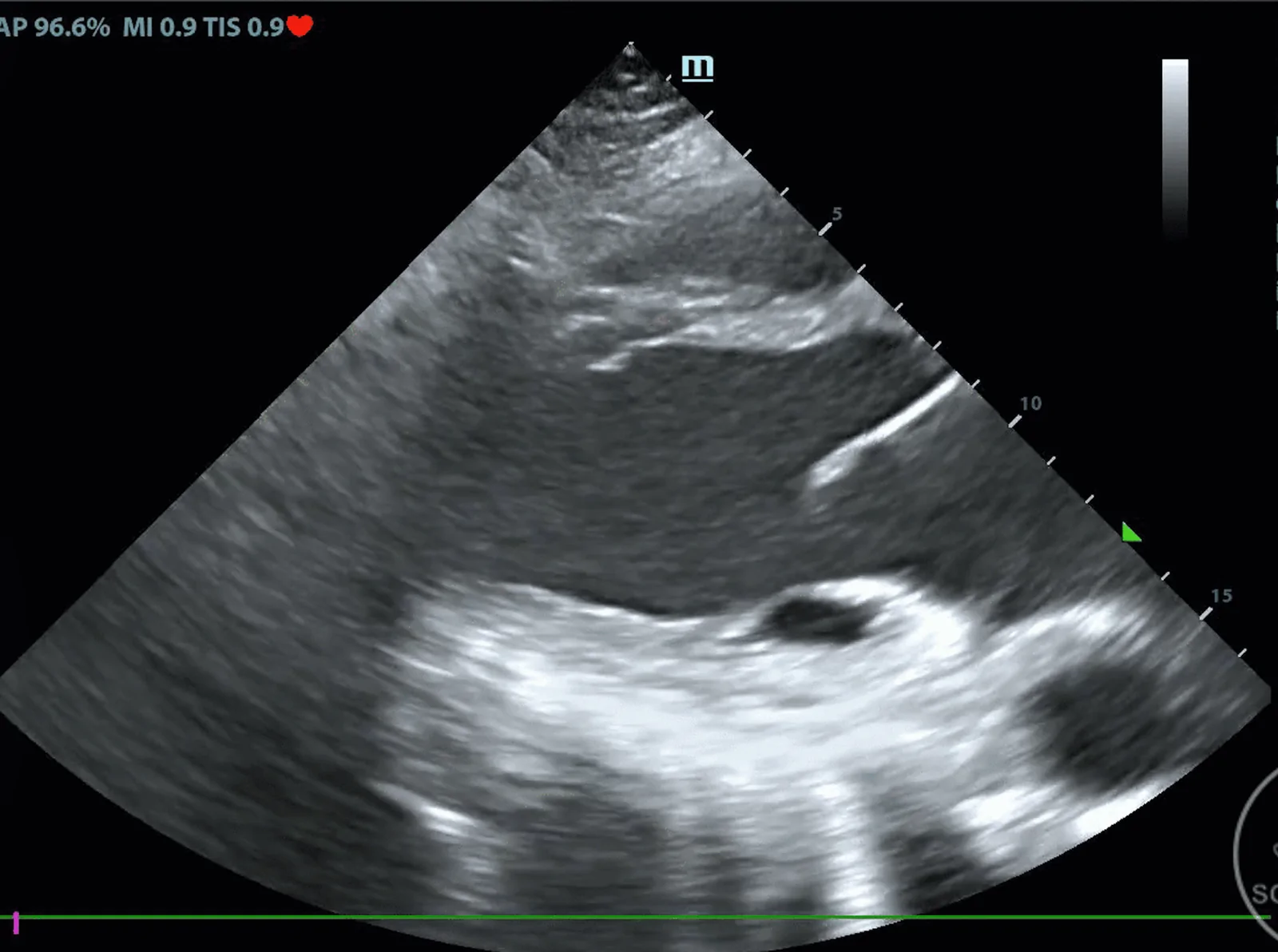
Cardiac ultrasound demonstrating left ventricular dysfunction

In this case, the video clip was an ultrasound of a heart in the parasternal long-axis view that demonstrated moderate left ventricular dysfunction. Examinees were asked to view the clip and identify artifacts, anatomy, or pathology that would be relevant for the complaint of shortness of breath. Finally, after interpreting the image, examinees were asked the following question:

“Assuming you saw diffuse B-lines on pulmonary ultrasound, in addition to the cardiac ultrasound you’ve seen already, what would you think about fluid administration for this patient who is hypotensive?”

After providing a response, the conclusion of the case was announced. The examinee left to another room to complete a post-survey and the proctor completed the grading form for this case.

Case 2: Thoracic ultrasound

Case 2 assessed the examinee’s ability to perform a thoracic ultrasound and assess a patient presenting with shortness of breath and right sided chest pain. The proctor was provided with a script (Appendix 4), which guided the resident to demonstrate how they would assess dyspnea using ultrasound to evaluate the lungs, starting with the left lung. Residents were asked to state their probe of choice. When obtaining their images on the standardized patient, the examinees were prompted to ask the proctor to adjust gain and probe settings as needed. Examinees were asked to state when they felt they had obtained an acceptable view of the left lung to evaluate for shortness of breath on the standardized patient and display for the proctor to assess. Examinees were asked to identify several anatomic structures on this image (Appendix 2). Additionally, they were asked to identify the pleura and obtain a view of this region using M-mode. Examinees were then asked to direct their attention to an alternate screen in the room that displayed a video clip and still image with lack of lung sliding and “barcode” sign on M-mode, respectively, indicating pneumothorax (Figure 2).

**Figure 2:**
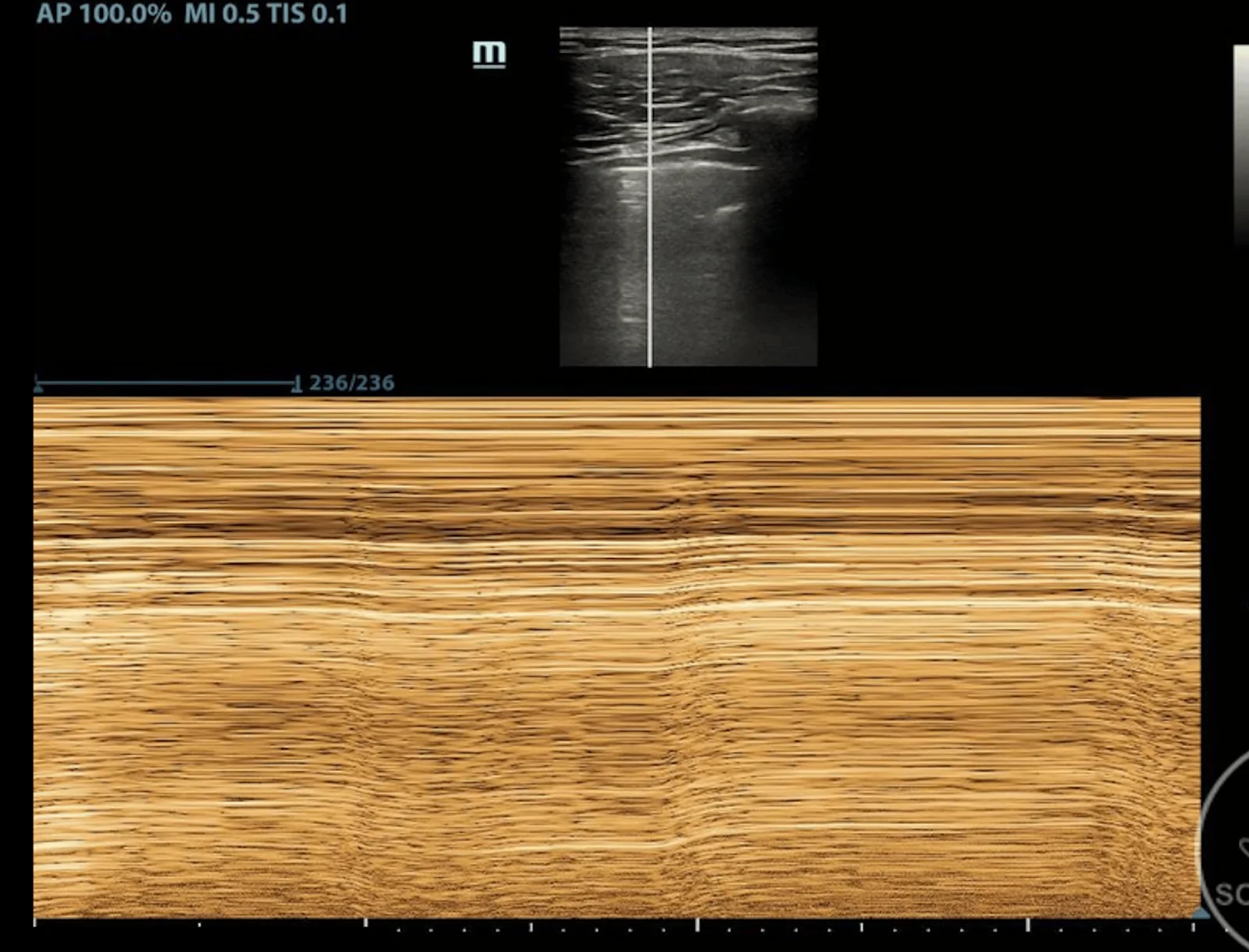
Ultrasound image using M-mode depicting “barcode sign” in pneumothorax

Examinees were asked to view the clip and identify artifacts, anatomy, or pathology that would be relevant for the complaint of shortness of breath and right sided chest pain. Finally, after interpreting the images, examinees were asked to suggest appropriate subsequent management of the patient. After providing a response, the conclusion of the case was announced. The examinee left to another room to complete a post-survey and the proctor completed the grading form for the case.

Data were summarized using Microsoft Excel 365. Statistical analyses were performed using IBM SPSS Statistics for Windows, version 30.0 (IBM Corp., USA). Demographic data were summarized using frequencies and percentages. Continuous variables were reported as means ± standard deviations or medians with interquartile ranges (IQRs), as appropriate. Independent-samples t-tests were used to compare mean examination scores between case groups and testing periods. Pearson correlation was used to evaluate the relationship between postgraduate year (PGY) and examination score.

An exploratory analysis compared examination scores between participants who self-reported reviewing the supplemental preparatory materials and those who did not. Because review of the supplemental materials was voluntary rather than randomized, this analysis was considered exploratory and was not interpreted as demonstrating a causal effect of the materials. Scores between these groups were compared using a two-sided Mann-Whitney U test and are reported as medians with IQRs. The Hodges-Lehmann estimator was used to describe the between-group location shift with a 95% confidence interval (CI). All statistical tests were two-tailed, with statistical significance defined as p < 0.05.

## Results

A total of 64 EM residents participated in the study. 48 completed the cross-over design, and 16 completed one day of the examination. Table 1 showcases examinee demographics, with post-graduate year, estimated prior ultrasound experience, and whether they reviewed the official ABEM certification ultrasound video.

**Table 1:** Examinee demographics PGY: Postgraduate year; ABEM: American Board of Emergency Medicine

Parameter	Category	Number (n)	Percentage (%)
PGY	PGY-1	23	36
	PGY-2	21	33
	PGY-3	20	31
Estimated prior ultrasound examinations performed	0–50	13	20
	51–100	7	11
	101–150	19	30
	151–200	11	17
	>200	14	22
Viewed ABEM instructional video	No	44	69
	Yes	20	31

In an exploratory analysis, 42 participants who self-reported reviewing the supplemental materials were compared with 17 participants who reported not reviewing the materials. The median examination score was 13 (IQR, 11-14.75) among participants who reviewed the supplemental materials and 13 (IQR, 13-14) among those who did not. A two-sided Mann-Whitney U test demonstrated no statistically significant difference between groups (U=301.5, p=0.35). The Hodges-Lehmann estimate of the location shift was 0 points (95% CI, approximately −2 to 0).

Examination performance

Mean examination scores for both exam type and exam day are shown in Table 2. On Day 1, the mean overall score (out of 15) was 11.96 ± 2.49 for the Cardiac group and 11.62 ± 1.92 for the Pulmonary group. On Day 2, the mean overall scores increased to 12.55 ± 2.45 and 12.75 ± 2.24, respectively. Independent-samples t-tests revealed no significant difference in mean scores between the Cardiac and Pulmonary cases on either Day 1 (p=0.57) or Day 2 (p=0.74). When comparing performance across days within each case type, Cardiac case scores showed a nonsignificant improvement (p=0.37), whereas Pulmonary case scores demonstrated a statistically significant increase from Day 1 to Day 2 (p=0.05).

**Table 2:** Mean performance scores and confidence ratings by case and training Day Independent-samples t-tests were used to calculate p-values.

Day	Case	Overall Score (Mean ± SD)	Initial Confidence (Mean ± SD)	Post-Test Confidence (Mean ± SD)	t-value	p-value
Day 1	Case 1 – Cardiac	11.96 ± 2.49	3.19 ± 1.14	3.81 ± 1.33	t=0.57	0.57
	Case 2 – Pulmonary	11.62 ± 1.92	3.12 ± 0.91	3.81 ± 0.94		
Day 2	Case 1 – Cardiac	12.55 ± 2.45	3.06 ± 0.96	3.68 ± 1.05	t=-0.33	0.74
	Case 2 – Pulmonary	12.75 ± 2.24	3.64 ± 0.99	3.93 ± 1.02		

Self-reported confidence had a mixed trend. Mean initial confidence on Day 1 was 3.19 ± 1.14 for the Cardiac group and 3.12 ± 0.91 for the Pulmonary group, and on Day 2 3.06 ± 0.96 and 3.64 ± 0.99, respectively. Mean post-test confidence was 3.81 ± 1.33 (Cardiac) and 3.81 ± 0.94 (Pulmonary) on Day 1, changing to 3.68 ± 1.05 and 3.93 ± 1.02 on Day 2. Overall, while both groups demonstrated improvement in performance over time, only the Pulmonary case group achieved a statistically significant gain in mean examination score between testing days.

Across all participants on Day 1, the PGY level was moderately correlated with total score (r=0.45, p=0.0007), with more senior residents tending to achieve higher examination scores. Among participants on Day 1, those who viewed the ABEM certification ultrasound example video scored 11.67 ± 2.18, compared to 11.82 ± 2.24 among those who did not. An independent-samples t-test showed no statistically significant difference in total score between these groups (t=−0.19, p=0.85).

## Discussion

The maturation of POCUS within the specialty of EM has led to its incorporation into the newest iteration of the ABEM Certifying Examination. This change reflects the reality of modern emergency practice, where bedside ultrasound is now routinely integrated into diagnostic evaluation, procedural guidance, and time-sensitive clinical decision-making [5]. As ultrasound has evolved from an adjunctive skill held by few to a core competency for graduating residents, assessment of these abilities during board certification represents a logical progression in competency-based evaluation.

The updated certifying exam may have several aspects that examinees will be unfamiliar with, such as the need to verbalize image acquisition and interpretation in real time. Examinees must not only obtain adequate images but also describe anatomic structures, recognize artifacts or pathology, and communicate their reasoning aloud. In addition, candidates must direct the examiner in manipulation of the ultrasound machine, including probe selection, preset choice, gain, depth, and other image optimization settings. The standardized patient format also introduces an element of patient-centered communication and professionalism, requiring examinees to interact appropriately while performing the examination. This combination of technical performance, communication, and clinical reasoning resembles an OSCE model and differs from the traditional written or oral assessments. While there is an abundance of literature available on how to train residents on POCUS, there is less evidence on how to train them for this specific testing environment [6].

This style of ultrasound assessment is consistent with approaches used in other specialties. For example, the American Board of Anesthesiology certifying examination includes OSCE components that assess image interpretation and ultrasound-guided clinical skills, while critical care and pulmonary training programs increasingly include ultrasound milestones and procedural competency assessments [7,8]. The incorporation of ultrasound into high-stakes certification examinations across specialties highlights the growing expectation that physicians demonstrate bedside imaging competence in a standardized manner.

The findings of this study suggest that EM residents may benefit from exposure to this format prior to the certifying examination. Resident performance improved over time, with statistically significant gains observed in the pulmonary case group and non-significant upward trends in the cardiac group. These findings indicate that repeated exposure to mock ABEM-style ultrasound cases may increase familiarity with the examination format and may be associated with improved performance on subsequent assessments. Although the supplemental study materials were associated with higher median scores among participants who reviewed them, this difference did not reach statistical significance. This may reflect limited sample size, variation in time spent reviewing the materials, or differing baseline ultrasound experience among participants. Nonetheless, the direction of effect suggests that concise, targeted review resources may still provide educational benefit.

PGY was moderately correlated with examination performance, with more senior residents achieving higher scores. This is not unexpected, as advancing trainees generally have greater cumulative clinical exposure, more independent ultrasound scanning experience, and more opportunities to synthesize ultrasound findings into medical decision making. Importantly, however, residents at all training levels were able to participate successfully, suggesting that structured curricula may be useful throughout residency training rather than reserved only for senior learners preparing for graduation.

Confidence scores demonstrated a mixed pattern. While many participants reported increased confidence after case completion, improvements in confidence did not always parallel objective performance gains. This finding is educationally relevant, as perceived competence and measured competence are not synonymous. Simulation-based or OSCE-style practice may therefore serve a dual purpose: improving technical readiness while helping learners calibrate self-assessment before a high-stakes examination.

These results have several implications for residency education as they support the claim that programs should consider incorporating mock ultrasound OSCEs into existing ultrasound curricula. Prior to this year, EM resident ultrasound curriculum was largely based on the ultrasound guidelines put forth in a policy statement by the American College of Emergency Physicians [5]. Much of the training focused on image acquisition and identification of pathology. However, to be successful on the new certifying exam, training should emphasize not only image acquisition and interpretation, but also verbalization of findings, machine optimization commands, and professional interaction with standardized patients. Our study demonstrates that introducing brief, high-yield cases with a standardized patient may be a practical and scalable intervention for residency programs seeking to prepare trainees for the new ABEM format without extensive curricular burden.

This study has several limitations. Participation was voluntary, introducing potential selection bias toward residents with greater interest or confidence in ultrasound. Although conducted at multiple residency programs, the sample size was modest, and not all participants completed the crossover design. It is unclear was there was significant attrition in study participants after completing the first study case. The study used locally developed mock cases that were modeled after available ABEM sample materials but cannot perfectly replicate the actual certifying examination. Examiner scoring, while standardized through rubrics, may still have been subject to inter-rater variability. Additionally, long-term retention of skills and direct correlation with future board examination success were not assessed. Although a one-month period was incorporated between examinations and participants crossed over to a different ultrasound case, sequence and carryover effects were not formally evaluated. Therefore, a learning or practice effect related to prior exposure to the examination format cannot be excluded and may have contributed to improved performance on the second examination.

There are also some limitations the chosen methods of analysis. The statistical analysis did not formally model participant, period, sequence, or carryover effects inherent to the crossover design. Additionally, because participants completed alternate cases at the second assessment, case-specific comparisons between Day 1 and Day 2 represent comparisons between different groups of participants rather than within-participant changes on the same case. These findings should therefore be interpreted as differences in case performance across testing periods rather than longitudinal improvement in performance on a specific ultrasound examination.

Future studies would benefit from identifying residency programs that have incorporated this OSCE-style ultrasound training into their curriculum and track certifying exam results, comparing outcomes to programs that do not teach to this component of the exam.

## Conclusions

A focused, two-case preparatory curriculum was feasible across multiple residency programs and associated with differences in performance across testing days, particularly in the pulmonary ultrasound scenarios. Early exposure to mock examination cases may help residents become familiar with the OSCE-style interface and practice the technical, interpretive, and communication skills required by the new examination format. Further controlled studies are needed to determine whether such preparation translates into improved performance on standardized or certifying examinations. As the new examination format is implemented, residency programs should consider structured ultrasound preparation as part of comprehensive board readiness training.

## Appendices

Appendix 1: Case 1 grading criteria

Patient-Centered Care

1)  Introduce yourself to the patient and wear gloves.

2)  Inform the patient of cold gel before application.

3)  Warn the patient of pressure when applying the probe for cardiac ultrasound.

Image Acquisition - Cardiac US

4)  Probe selection - Phased array

5)  Depth - Appropriate to visualize descending aorta or 1 cm deeper than pericardium

6)  Gain - Appropriate to visualize endocardial border OR valvular function

7)  Quality - Appropriate to visualize left ventricular function, including mitral valve motion

Image Interpretation - Cardiac US

8)  Anatomy - Appropriately identified left ventricle

9)  Anatomy - Appropriately identifies descending aorta

10)  Anatomy - Appropriately identifies left atrium

11)  Anatomy - Appropriately identifies mitral valve

12)  Anatomy - Appropriately identifies right ventricle

13)  Anatomy - Appropriately identifies aortic valve/left ventricular outflow track

14)  Application - Appropriately identifies decreased ejection fraction/heart failure/decreased left ventricular function

15)  Application - Considers vasopressors or restrictive IVF administration in the setting of diffuse B lines and decreased ejection fraction

Appendix 2: Case 2 grading criteria

Patient-Centered Care

1)  Introduce yourself to the patient and wear gloves. 

2)  Inform the patient of cold gel before application. 

3)  Warn the patient of pressure when applying the probe. 

Image Acquisition - Thoracic Examination

4)  Probe selection - linear probe

5)  Depth - Appropriate to the pleura, without excessive depth (no more than 7cm deep to pleura)

6)  Gain - Appropriate to visualize lung sliding

7)  Quality - Appropriate to visualize lung sliding

Image Interpretation - Cardiac US

8)  Anatomy - Identifies pleural sliding 

9)  Anatomy - Identifies ribs

10)  Anatomy - Identifies soft tissue (muscle and/or skin)

11) Anatomy - Identifies lung parenchyma/lung tissue

12)  Anatomy - Identifies sea-shore sign on M-mode

13) Application - Appropriately identifies lack of lung sliding indicating pneumothorax

14) Application - Appropriately identifies the bar-code sign on M-mode as indication pneumothorax

15) Application - Suggests appropriate subsequent management of the patient (oxygen, needle decompression, thoracostomy or chest tube)

Appendix 3: Case script 1

Case 1 Setup

Ultrasound Preparation:

Ensure phased array, linear, and curvilinear probes are present, functioning and clean.

Ensure the ultrasound screen is easily visible to the examinee, either directly or via screen share.

Please complete the pre-test survey questions.

When Examinee Enters:

“Hello Doctor, I am Dr. _ and will be assisting as your examiner for this ultrasound case. As a reminder, please ask me to change probes, change depth, gain, or mode of ultrasound to assist you with the case. Please ask for help to reposition the patient or bed. Do you have any questions?”

Case Vignette:

“As you have noted, the patient is a 54-year-old male presenting with progressive shortness of breath.”

Cardiac Ultrasound:

“I would like for you to now demonstrate how you would assess dyspnea using ultrasound to evaluate left ventricular function with a parasternal long-axis view. Which probe would you like to use?”

Probe Settings:

Linear: Gain 10, depth 10cm

Phased array: Gain 10, depth 5cm

Curvilinear: Gain 10, depth 5cm

If Asked to Change Probe Settings:

“Tell me when to stop adjusting [gain/depth]."

Adjust the gain and depth slowly, allowing the examinee to stop you.

“When you find an acceptable image, please let me know.”

After Examinee Has Selected Their Image:

“Thank you, I will take the probe from you now. Please direct your attention to the screen. Assume this video is the video you obtained from this patient. I would like you to point out any artifacts, anatomy, or pathology that would be important for this complaint.”

After interpreting the normal images, direct the examinee to the screen where you will show them pathology: heart dysfunction/heart pathology clip

“Assuming this is your patient’s ultrasound, please interpret the image/clip, pointing out any pathology.”

Application:

“Assuming you saw diffuse B-lines on pulmonary ultrasound, in addition to the cardiac ultrasound you’ve seen already, what would you think about fluid administration for this patient who is hypotensive?”

End of Case:

“Thank you, that concludes your case.”

Please complete the post survey questions

Appendix 4: Case script 2

Ultrasound Preparation

Ensure phased array, linear, and curvilinear probes are present, functioning and clean.

Ensure the ultrasound screen is easily visible to the examinee, either directly or via screen share.

Please complete the pre-test survey questions

When Examinee Enters

“Hello Doctor, I am Dr. _ and will be assisting as your examiner for this ultrasound case. As a reminder, please ask me to change probes, change depth, gain, or mode of ultrasound to assist you with the case. Please ask for help to reposition the patient or bed. Do you have any questions?”

Case Vignette

“As you have noted, the patient is a 48 year-old male presenting with shortness of breath and right sided chest pain.”

Thoracic Ultrasound

“I would like for you to now demonstrate how you would assess dyspnea using ultrasound to evaluate the lungs, starting with the left lung. Which probe would you like to use?”

Probe Settings

Linear: Gain 10, depth 10 cm

Phased array: Gain 10, depth 5 cm

Curvilinear: Gain 10, depth 5 cm

If Asked to Change Probe Settings

“Tell me when to stop adjusting [gain/depth].”

Adjust the gain and depth slowly, allowing the examinee to stop you.

“Please assess the left lung now. When you find an acceptable image, please let me know.”

After Examinee Has Selected Their Image

“Thank you, I will take the probe from you now. Please direct your attention to the screen. Assume this video is the video you obtained from this patient. I would like you to point out any artifacts, anatomy, or pathology that would be important for this complaint.”

“Thank you, now please obtain an M-mode image of the pleura”

After interpreting the normal images, direct the examinee to the screen where you will show them pathology: Clip demonstrating lack of lung sliding and "barcode sign" image

“Assuming this is your patient’s right lung ultrasound, please interpret the image/clip, pointing out any pathology.”

Application

"Assuming there was an absence of lung sliding, and CXR showed 40% collapse of the right lung, what would be your next steps in managing the patient?”

*End of Case* 

“Thank you, that concludes your case.”

Please complete the post survey questions.

## References

[1] (2026). Certifying Exam. https://www.abem.org/get-certified/certifying-exam/.

[2] Haidar DA, Peterson WJ, Minges PG (2022). A consensus list of ultrasound competencies for graduating emergency medicine residents. AEM Educ Train.

[3] Nelson M, Abdi A, Adhikari S (2016). Goal‐directed focused ultrasound milestones revised: a multiorganizational consensus. Acad Emerg Med.

[4] (2026). New certifying exam resources available. https://www.abem.org/news/new-certifying-exam-resources-available/.

[5] (2023). Ultrasound guidelines: emergency, point-of-care, and clinical ultrasound guidelines in medicine. Ann Emerg Med.

[6] PoSaw LL, Wubben BM, Bertucci N, Bell GA, Healy H, Lee S (2021). Teaching emergency ultrasound to emergency medicine residents: a scoping review of structured training methods. J Am Coll Emerg Physicians Open.

[7] (2026). Objective Structured Clinical Examination (OSCE) Content Outline. American Board of Anesthesiology.

[8] (2026). ACGME Program Requirements for Graduate Medical Education in Pulmonary Disease and Critical Care Medicine Including FAQs. https://www.acgme.org/globalassets/pfassets/programrequirements/2026-prs/156_pulmonarydiseasecriticalcaremedicine_2026.pdf.

